# Extraction of a Novel Cold-Water-Soluble Polysaccharide from *Astragalus membranaceus* and Its Antitumor and Immunological Activities

**DOI:** 10.3390/molecules23010062

**Published:** 2017-12-28

**Authors:** An-jun Liu, Juan Yu, Hai-yu Ji, Hong-cui Zhang, Yan Zhang, Hui-ping Liu

**Affiliations:** Key Laboratory of Food Nutrition and Safety, Ministry of Education, College of Food Engineering and Biotechnology, Tianjin University of Science and Technology, Tianjin 300457, China; laj1456@163.com (A.L.); yujuan14615@163.com (J.Y.); haiyu11456@163.com (H.J.); jihaiyu1247@163.com (H.Z.); jhy1247@163.com (Y.Z.)

**Keywords:** *Astragalus membranaceus*, cold-water-soluble polysaccharide, structure characterization, immunomodulation, antitumor

## Abstract

The polysaccharides of *Astragalus membranaceus* have received extensive study and attention, but there have been few reports on the extraction of these polysaccharides using cold water (4 °C). In this study, we fractionated a novel cold-water-soluble polysaccharide (cAMPs-1A) from *Astragalus membranaceus* with a 92.00% carbohydrate content using a DEAE-cellulose 52 anion exchange column and a Sephadex G-100 column. Our UV, Fourier-transform infrared spectroscopy (FTIR), high-performance gel permeation chromatography, and ion chromatography analysis results indicated the monosaccharide composition of cAMPs-1A with 1.23 × 10^4^ Da molecular weight to be fucose, arabinose, galactose, glucose, and xylose, with molar ratios of 0.01:0.06:0.20:1.00:0.06, respectively. The UV spectroscopy detected no protein and nucleic acid in cAMPs-1A. We used FTIR analysis to characterize the α-d-pyranoid configuration in cAMPs-1A. In addition, we performed animal experiments in vivo to evaluate the antitumor and immunomodulatory effects of cAMPs-1A. The results suggested that cAMPs-1A oral administration could significantly inhibit tumor growth with the inhibitory rate of 20.53%, 36.50% and 44.49%, respectively, at the dosage of 75,150, and 300 mg/kg. Moreover, cAMPs-1A treatment could also effectively protect the immune organs, promote macrophage pinocytosis, and improve the percentages of lymphocyte subsets in the peripheral blood of tumor-bearing mice. These findings demonstrate that the polysaccharide cAMPs-1A has an underlying application as natural antitumor agents.

## 1. Introduction

Traditional Chinese medicines (TCMs), known to be effective agents in the early prevention and treatment of certain diseases, are being considered as a new source of anticancer agents and chemotherapeutic adjuvants to strengthen the efficacy of chemotherapy and mitigate its toxic effects [1]. *Astragalus membranaceus* (also known as Huangqi in Chinese) is the dry roots of *Astragalus Mongholicus Bunge*, which has shown strengthening and restoring effects and is a herbaceos perennial of the Leguminosae that is extensively distributed throughout the temperate regions of the world [2,3]. There are more than 100 chemical compounds in the roots of *A. membranaceus*, including polysaccharides, flavonoids, astragalosides, amino acids, and other trace elements [4]. In the last two decades, researchers have gained insight into the pharmacological functions of these components, especially the polysaccharides extracted from *A. membranaceus*. Recently, *A. membranaceus* polysaccharides have been reported to be characterized by various biological activities, including antitumor, immunomodulatory, antioxidant, antidiabetes, antimicrobial, and antiinflammation activities [5,6]. Zhu et al. obtained anti-tumor polysaccharides from *Astragalus* mainly composed of α-(1→3) glucose and a few 1→4, 1→6 glucoses in main chains, and arabinoses and xyloses in the side chain [7]. Rui et al. isolated the *Astragalus* polysaccharides as an α-(1→4)-d-glucan with α-(1→6)-linked branches attached to the O-6 of branch points, which exhibited immune modulating activities in rats [8]. Yan et al. extracted polysaccharides from *A. membranaceus* that mainly consisted of mannose, glucose and arabinose with the presence of polyphenols and furan ring, which showed significant antioxidant activity in mice [9].

Hepatocellular cancer is a major health problem in Asia with a high incidence and rate of mortality [10] and although modern cancer therapies have been greatly improved, it is still difficult to take effective action during the terminal stage of cancer. At present, more attention is being given to finding effective strategies for completely eliminating tumor cells, such as vaccinotherapy [11] and immunotherapy [12]. Although, the security of effective vaccination makes it worthy of consideration, it will be very difficult to make this approach effective for the treatment of cancers generally. At the same time, *A. membranaceus* polysaccharides have been reported to have antitumor and immunoregulation bioactivities, and the qualities of these activities are inextricably linked with their chemical constituents, structure, and molecular weight, as well as their physical properties [13].

Currently, the most common polysaccharide extraction method is hot-water extraction and alcohol sedimentation, although the pharmacological activity may be decreased under the high extractive temperatures used (commonly 80–100 °C), which likely results in the degradation [14] and oxidation [15] of the polysaccharides. Cho et al. investigated the in vitro and in vivo antitumor effects of five bioactive fractions isolated from *A. membranaceus* with a 95 °C extraction temperature and stepwise ethanol precipitations. The results demonstrate that A1, the most valuable component, suppressed by less than 45% the proliferation of most tumor cells (J774 A.1, PU5-1.8, HL-60, M1, L929, and WEHI-164) when co-cultured for 48 h at a concentration of 200 µg/mL [2]. In our preliminary experiments, *A. membranaceus* polysaccharides extracted at 95 °C showed approximately 30% suppression of H22 cell proliferation when co-cultured for 48 h at a concentration of 200 µg/mL, whereas the polysaccharides extracted at 4 °C showed more than 60% suppression under the same conditions. Therefore, to ensure higher levels of bioactivity, the scientific community should develop more efficient extraction methods for *A. membranaceus* polysaccharides. The ideal extraction method should ensure higher polysaccharide bioactivity and relatively lower energy consumption. 

There are few reports on the extraction of the cold-water-soluble *A. membranaceus* polysaccharide (cAMPs) and its antitumor and immunoregulation activities. In this study, we isolated and purified a novel cold-water-soluble polysaccharide (cAMPs-1A) from *A. membranaceus* and evaluated its structure and in vivo antitumor activity. This study offers new ideas for producing more reasonable and effective anticancer drugs from TCM.

## 2. Results

### 2.1. Isolation, Purification and Chemical Composition Analysis

We obtained the crude polysaccharide (cAMPs) by cold-water (4 °C) extraction, 80% alcohol precipitation, and deproteinization with a yield of 5.15%, which we calculated using the weight of the dried roots of *A. membranaceus*. We further fractionated and purified the cAMPs in a DEAE-cellulose 52 anion exchange column and a Sephadex G-100 column to obtain the major peak eluted with distilled water (cAMPs-1A), with a yield of 37.5%, based on the weight of the cAMPs.

The total carbohydrate content in cAMPs-1A was about 92.00%, based on our results using the phenol-sulfuric acid method, and the protein content was 0.11%. Meanwhile, we detected little uronic acid (0.24%) in cAMPs-1A by the carbazole-sulfuric acid method, which implies that it was a neutral polysaccharide. The specific optical rotation of cAMPs-1A, which we determined at 25 °C using an automatic polarimeter (Model WZZ-2B, Shanghai, China), was +176°. The positive values indicate that the glycosidic bond of cAMPs-1A can be categorized as α-type.

### 2.2. HPGPC and IR Analyses

**Figure 1:**
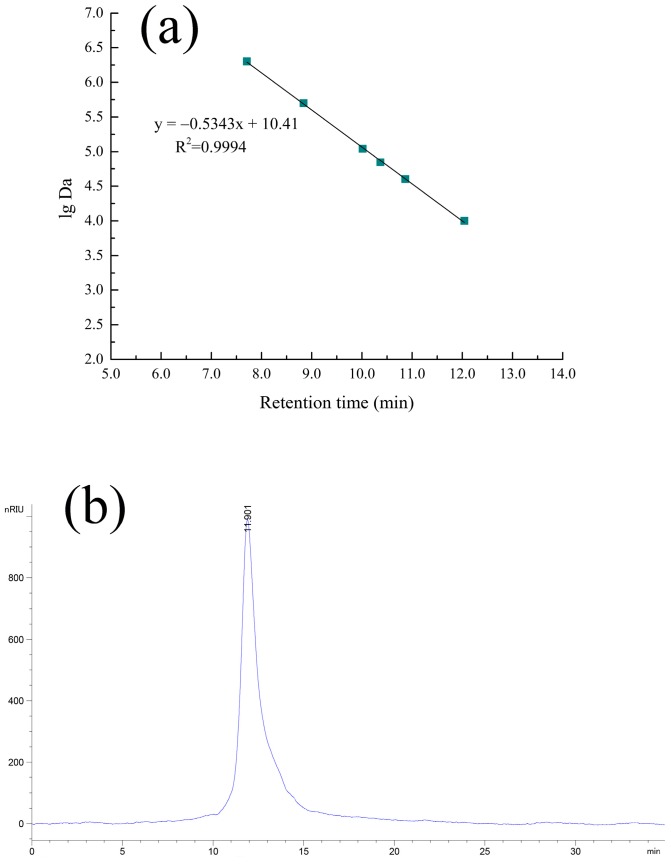
The standard curve (**a**) and results of HPGPC of cAMPs-1A (**b**).

As shown in Figure 1a, the calibration curve of serial Dextran standards was plotted as the molecular weight on a log scale (lg Da) versus the retention time (R_t_). The regression equation was y = −0.5343x + 10.41, R^2^ = 0.9994 (y = lg Da, x = R_t_). As seen from Figure 1b, the HPGPC profiles of cAMPs-1A had only one symmetrical narrow peak, which suggested that cAMPs-1A was a homogeneous polysaccharide. Based on the standard dextran curve, the average molecular weight of cAMPs-1A was calculated to be 1.23 × 10^4^ Da.

**Figure 2:**
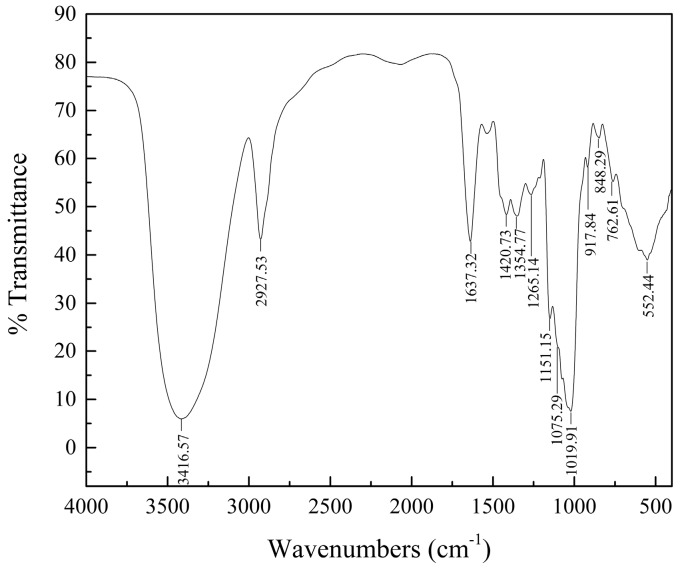
Results of IR spectrum on cAMPs-1A.

The IR spectra of cAMPs-1A (Figure 2) reveals that these polysaccharides have three characteristic absorption peaks: an intense peak at 3416.57 cm^−1^, which we can attribute to the -OH stretching vibration, a weak peak at 2927.53 cm^−1^ due to the C-H stretching vibration, and a peak at 1637.32 cm^−1^ due to the C=O stretching vibration [16]. Each polysaccharide has a specific bond in the region of 1200–1000 cm^−1^, which is considered to be the fingerprint region, and which implies that the polysaccharide characteristics accord with the position and intensity of the bonds [17]. We can assign the three characteristic bands at 1151.15, 1075.29 and 1019.91 cm^−1^ to the asymmetric vibrations of C-O-C glycosidic rings, which suggests the presence of pyranose in cAMPs-1A [18]. We ascribe the band at 848.29 cm^−1^ to the α-type glycosidic linkages in its structure. The bands at 848.29 and 917.84 cm^−1^ are characteristic of (1→4)-α-glucan [19], which differed slightly from a previous study. 

### 2.3. Monosaccharide

**Figure 3:**
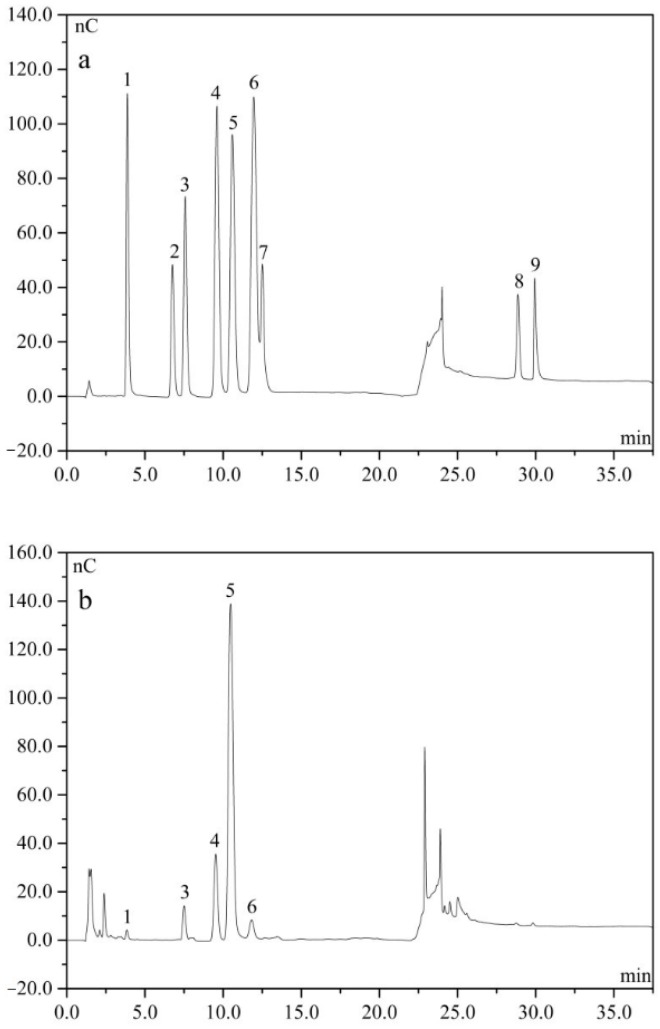
Ion chromatograms of standard monosaccharide (**a**) and cAMPs-1A (**b**). Peak identity: 1—fucose, 2—rhamnose, 3—arabinose, 4—galactose, 5—glucose, 6—xylose, 7—mannose, 8—glucuronic acid, 9—galacturonic acid.

We determined the monosaccharide composition and molar ratios of the cAMPs-1A by IC. Our monosaccharide component analysis indicated that the monosaccharide composition of cAMPs-1A mainly consisted of glucose, which accounted for 75.19% of all the quantified monosaccharides, with fucose, galactose, arabinose, and xylose also present in small amounts (Figure 3). In other reports, the monosaccharide residues and molar ratios of polysaccharide fractions isolated from *A. membranaceus* by other extraction methods were significantly more diverse and fucose was a sort of rare monosaccharide in cAMPs-1A [15]. We found no uronic acid in the cAMPs-1A, which indicates that it was a neutral polysaccharide and which supports the results of our chemical composition analysis.

### 2.4. Antitumor Activity of cAMPs-1A In Vivo

As shown in Table 1, there was an obvious increase in the spleen index and a significant decrease in the thymus index in the model group compared to the blank group (*p* < 0.05), whereas there was a notable decrease in the spleen index and an increase in the thymus index in the cAMPs-1A groups compared to the model group (*p* < 0.05). Indeed, the thymus and spleen indexes of the cAMPs-1A group with a high dose (300 mg/kg) reached the level of the blank group, which suggested that cAMPs-1A might protect the immune organs of tumor-bearing mice from serious damage in a dose-dependent manner. 

The tumor weight in the model group was 2.63 ± 0.24 g after 15 days inoculation with H22 cells. As compared with the model group, the weights of the transplanted tumors in the polysaccharides groups (75, 150 and 300 mg/kg) were significantly decreased (*p* < 0.05), with inhibitory ratios of 20.53%, 36.50%, and 44.49%, respectively. As shown in Table 1, mice treated with 5-fluorouracil (5-FU) which was the main chemotherapy drug for clinical tumors [20] exhibited the highest antitumor ability, with an inhibitory rate of 46.39%, however, the thymuses and spleens were also impaired by this chemotherapeutic agent.

### 2.5. Effects of cAMPs-1A on the Macrophage Pinocytosis and NK Cytotoxicity

**Figure 4:**
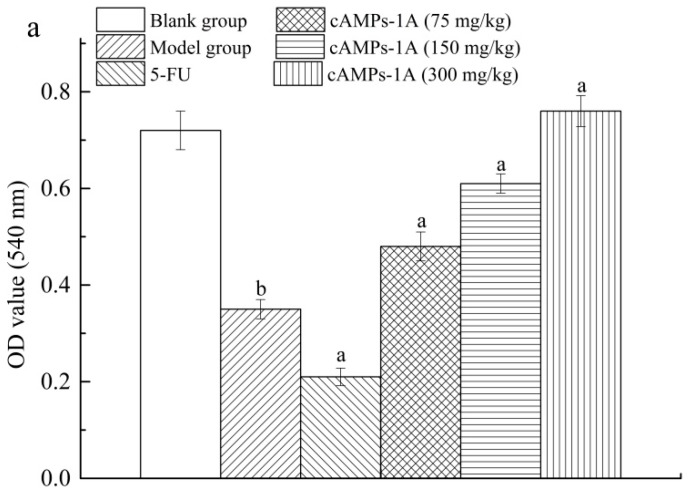

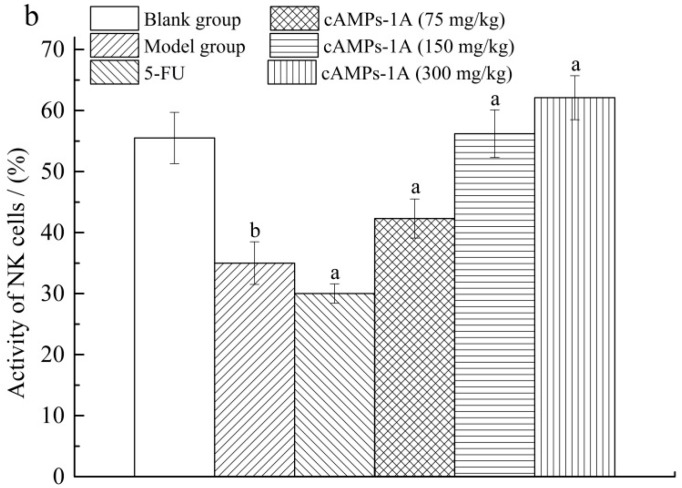
Effects of cAMPs-1A on macrophages pinocytosis (**a**) and NK cells activity (**b**) in H22-bearing mice. Note: ^a^ *p* < 0.05 compared to model group; ^b^ *p* < 0.05 compared to blank group.

Macrophages are known to be an important class of immune cells of the host defense against invading pathogens and tumor growth [21]. As shown in Figure 4a, the phagocytic function of intra-abdominal macrophages in the model group is decreased compared to that of the blank group (*p* < 0.05). The phagocytic function of the 5-FU treated group also notably decreased in contrast with the model group (*p* < 0.05). However, cAMPs-1A effectively improved the pinocytosis of the macrophage toward the neutral red as compared with the model group (*p* < 0.05) in a dose-related manner. These results indicate that cAMPs-1A might enhance macrophage pinocytosis and reduce the sacrifice of macrophages in the process of fighting against H22 tumor cells. 

As shown in Figure 4b, the killing activity of NK cells in the blank group was significantly higher than that of the model group (*p* < 0.05), whereas the 5-FU treated H22-bearing mice exhibited lower killing activity by NK cells than the model group (*p* < 0.05). However, the NK cells activity of the 75, 150, and 300 mg/kg cAMPs-1A groups were significantly enhanced in comparison with the model mice (*p* < 0.05), dose-dependently. Moreover, the macrophage pinocytosis and NK cytotoxicity in the 300-mg/kg cAMPs-1A group were considerable compared with those of normal mice.

### 2.6. Effects of cAMPs-1A on the T Cells Subsets in Peripheral Blood

**Figure 5:**
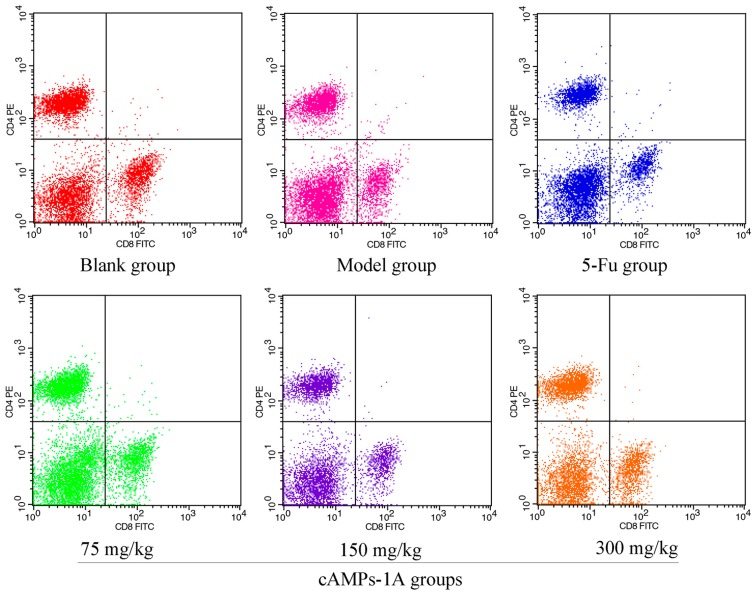
Effects of cAMPs-1A on T cells subsets distribution in peripheral blood of H22-bearing mice.

Lymphocytes have both regulatory and effector roles and the distribution of the different subsets in peripheral blood can change with the occurrence of cancer [22]. As shown in Table 2, there were significant decreases in the proportions of CD4^+^ and CD8^+^ T cells in the peripheral blood of tumor-bearing mice in the model group relative to the blank group (*p* < 0.05), which indicates that the immunologic balance of the tumor-bearing mice in the model group had been destroyed under the attack of the H22 tumor cells [23,24]. The proportions of CD4^+^ and CD8^+^ T cells in 5-FU and cAMPs-1A with 75 mg/kg showed no difference compared with those of the model group, whereas the higher doses of cAMPs-1A (150 mg/kg and 300 mg/kg) significantly increased the proportions of both the CD4^+^ and CD8^+^ T cells in peripheral blood (*p* < 0.05), especially the CD4^+^ T cells (Figure 5). These results suggested that cAMPs-1A could enhance the cellular immune functions of H22 tumor-bearing mice. 

## 3. Discussion

Recently, polysaccharides that can enhance the body’s immunity have attracted great interest with respect to the development of effective agents to treat cancer patients [25]. In our study, we extracted a novel cold-water-soluble polysaccharide (cAMPs-1A) from the roots of *A. membranaceus* and purified it using a DEAE-cellulose 52 anion exchange column and a Sephadex G-100 column. Our chemical analysis, HPGPC, IR, and IC results showed cAMPs-1A to be a neutral polysaccharide with a molecular weight of 1.23 × 10^4^ Da, which is a pyranose with α-type glycosidic linkages, and is mainly composed of glucose (75.19%) and small amounts of fucose, arabinose, galactose and xylose. Compared with the hot-water-extracted polysaccharide consisting of over 90% of glucose as their major sugar [7], our results demonstrated that cAMPs-1A was composed of various monosaccharides, which indicated that lower temperature might prevent the degradation of polysaccharide and assure its bioactivities.

As we all know, the antitumor and immunoregulatory activities of polysaccharides were related to their structure, monosaccharide composition, molecular weight and uronic acid content and so on [26]. In general, polysaccharides with pyranoid ring and good solubility will possess better immunoregulation effect in vivo [27,28]. Moreover, it was reported that α-configuration glycosidic bond in polysaccharides would also contribute to the antitumor activities [29]. In this study, cAMPs-1A exhibited α-type glycosidic linkages and pyranose form with good water solubility, and also showed good antitumor and immunoregulatory activities, which were consistent with previous researches.

Currently, chemotherapy remains the main treatment option for cancer patients, but the severe clinical side effects limit its therapeutic use [30,31]. In this study, we investigated the antitumor effects of cAMPs-1A on H22-bearing mice. We observed a significant decrease in the solid tumors of polysaccharide-treated mice compared with the model group, and the inhibitory rate reached to 44.49% in high-dose group. 5-FU showed the similar inhibition effect (inhibitory rate of 46.39%) on solid tumor, while the immune parameters were depressed, which indicated that chemotherapy could directly inhibit the proliferation of cancer cells but also adversely affects the functions of immune system. 

Macrophages and NK cells, which belong to the innate immune system, have the ability to rapidly respond to malignant cells [32,33]. Macrophages could phagocytose invading antigens and initiate the innate immune response via releasing tumor necrosis factor (TNF)-α, interleukin (IL)-6, IL-12, and nitric oxide (NO) [34,35]. NK cells are cytolytic effector lymphocytes that directly kill tumor cells and prevent pulmonary metastasis and peritoneal dissemination in the absence of specific immunization [36,37]. In the present work, cAMPs-1A could effectively improve the macrophages pinocytosis and NK cells killing activity, especially in the high-dose group, leading to the enhancement of antitumor immunity. 5-FU could directly induce both tumor cells and immune cells apoptosis, resulting in the immunosuppression of the host.

The thymus and spleen are important tumor-related immune organs [38]. Thymus provides a suitable microenvironment for T cells differentiation and maturation to CD4^+^ and CD8^+^ T cells [39]. Spleen plays a central role in immunoregulatory effect consisting of various immune cells including lymphocytes, monocytes and macrophages [40]. The cellular antitumor immune response could effectively eliminate tumor cells and mainly involves the capture and processing of tumor-derived antigens by dendritic cells, the recognition of CD4^+^ and the cytotoxicity of CD8^+^ T cells [41], all of which play central roles in the fight against tumors. In this work, we found that cAMPs-1A could significantly protect the immune organs (thymus and spleen) and suppress the growth of tumors, and improve the proportions of T cells subsets, dose-dependently, while 5-FU showed serious side effects on immune organs and the population of T cells, which indicated that cAMPs-1A exhibited strong antitumor and immunoregulatory activities in vivo with little side effect and had the potential to be developed as a novel antitumor agent.

## 4. Materials and Methods

### 4.1. Materials

We purchased dried roots of *A. membranaceus* of superior quality from Qi Jing Ltd. (Beijing, China) and H22 cells (murine hepatocarcinoma cells) from the Shanghai Institute of Biological Sciences at the Chinese Academy of Sciences (Shanghai, China). We purchased 3-(4,5-dimethyl-2-thiazolyl)-2,5-diphenyl-2-*H*-tetrazolium bromide (MTT), dimethyl sulfoxide (DMSO), DEAE-cellulose 52, Sephadex G-100, and galacturonic acid from Sigma (St. Louis, MO, USA). We purchased bovine serum albumin (BSA), T-series dextran, standard monosaccharides, glucuronic acid, and 5-Fu from Solarbio Co. Ltd. (Shanghai, China). All of the chemicals and agents were of analytical grade.

### 4.2. Extraction, Isolation and Purification of cAMPs

We soaked 100 g of *A. membranaceus* in 1 L of distilled water three times, once every two days, at 4 °C. We then merged and concentrated the filtrate to approximately 300 mL using the cryoconcentration method, and then removed the protein using the Sevag method [42]. After removing the Sevag reagents, we precipitated the concentrate by adding four volumes of anhydrous ethanol overnight followed by centrifugation at 2500 rpm for 20 min. Then, we re-dissolved in distilled water, concentrated, dialyzed (Mw 3500), and finally lyophilized the precipitate to yield the crude polysaccharide (cAMPs).

We dissolved the prepared cAMPs in distilled water and isolated it using a DEAE-cellulose 52 anion exchange column (2.6 cm × 30 cm) and a Sephadex G-100 column (1.6 cm × 35 cm) eluted with distilled water. Then, we combined the main peaks and concentrated and lyophilized the cAMPs to obtain a purified polysaccharide we refer to as cAMPs-1A.

### 4.3. Partial Characterization and Structure Analysis of cAMPs-1A

#### Chemical Composition Analysis

We measured the total carbohydrate content using the modified phenol-sulfuric acid method with d-glucose as the standard [43]. We determined the protein content by the Coomassie brilliant blue method using bovine serum albumin as the standard [44]. We quantified the uronic acid content by the carbazole-sulfuric acid method using galacturonic acid as the standard [45]. We recorded all UV spectrophotometer (UV-2500PC, Shimadzu, Kyoto, Japan) measurements in triplicate. 

### 4.4. HPGPC and IR Spectrum Assays

We determined the average molecular weight of the polysaccharide fraction using a high-performance gel permeation chromatograph (HPGPC) (Agilent-1200, Agilent, Santa Clara, CA, USA) equipped with a TSK-gel G4000PWxl column (7.8 mm × 300 mm, column temperature 30 °C) and a refractive index detector (detector temperature 35 °C) (Schambeck SFD GmbH, Bad Honnef, German). We analyzed the sample (20 µL) using ultrapure water as the mobile phase at 0.6 mL/min. We calculated the molecular weight of the polysaccharide fraction using the standard curve, which was established using the T-series dextrans (T-10, T-40, T-70, T-110, T-500, and T-2000) as standards. We used the KBr pellet method to record the IR spectrum of the polysaccharide fraction in the range of 4000–400 cm^−1^ in a Fourier-transform infrared spectrophotometer (Bruker VECTOR-22, Karlsruhe, Germany). The details of this procedure are as follows: we mixed 0.7 mg of the sample with 150 mg of dry KBr and pressed this mixture into a disk for infrared spectroscopic analysis.

### 4.5. Analysis of Monosaccharide Composition

We identified and quantified the monosaccharide composition of the polysaccharide fraction by ion chromatography (IC), which we performed on a Dionex ICS2500 chromatographic system (Sunnyvale, CA, USA) equipped with a Dionex pulsed amperometric detector with an Au electrode and an efficient anion exchange column of Dionex Carbopac PA20 column (150 mm × 3 mm) [46]. We used the following method to analyze the sample: we hydrolyzed 5 mg of the sample with 1 mL of 2 M trifluoroacetic acid (TFA) in sealed tubes at 110 °C for 3 h. After removing the TFA, we dissolved the hydrolysates in 1 mL of distilled water and then diluted this solution 10-fold for analysis. We maintained the column temperature at 30 °C and the injection volume was 1 mL. We then successively eluted the hydrolysates with NaOH (6 mM) and NaAC (100 mM) solutions at a flow rate of 0.45 mL/min. We evaluated the monosaccharide composition and percentages using d-fucose, d-rhamnose, l-arabinose, d-galactose, d-glucose, l-xylose, d-mannose, d-glucuronic acid and d-galacturonic acid as references.

### 4.6. Animals and Experimental Design

We purchased female BALB/c mice (6–8 weeks old) weighing 18–22 g from the Center of Experimental Animals of the Academy of Military Science (Beijing, China). We raised these animals under pathogen-free conditions at a controlled temperature (20–25 °C) and relative humidity (50 ± 5%). They were allowed free access to tap water and were fed with a standard pellet diet on a 12-h light/dark cycle throughout the experimental period. We conducted all experimental procedures that involved the animals in accordance with the principles of Laboratory Animal Care as approved by the Local Ethics Committee for Animal Care and Use at the Tianjin University of Science and Technology.

For the experiment, we randomly divided 60 BALB/c female mice into six groups, including a blank group, model group, cAMPs-1A groups (75, 150, and 300 mg/kg) and positive group (5-FU, 30 mg/kg). After being allowed to adapt to the environment for one week, we intragastrically administered cAMPs-1A to the mice in the cAMPs-1A groups at dosages of 75 mg/kg, 150 mg/kg, and 300 mg/kg, respectively, in a volume of 0.2 mL once per day, whereas the blank group and model group mice were administered a saline solution only at the same intervals. After 15 days of gavage, we subcutaneously injected 0.2 mL of H22 cell suspension (2 × 10^6^ cells/mouse) into the armpit of their right hind limbs except blank group. Then, we continued to intragastrically administer to all groups once daily for another 15 days, whereas we intraperitoneally administered 5-FU in the positive group once daily for 10 days.

### 4.7. Immune Organ Indexes and Tumor Weight

24 h after the last dose, we weighed and then sacrificed all the mice by cervical dislocation and then carefully dissected and weighed their tumors, spleens and thymuses. We used immune organ indexes of the spleens and thymuses to express the influence of the cAMPs-1A on the immune organs, using the following formula: the organ indexes (%) = average weight of organs/(average body weight) × 100% [47]. We measured the tumor inhibitory rate by the following formula: inhibitory rate (%) = [(M − T)/M] × 100, where T and M are the average tumor weights of the treated and model groups, respectively [48].

### 4.8. Macrophage Pinocytosis and NK Cytotoxicity Assays

We measured the phagocytic function of the peritoneal macrophage as has been described previously, with a slight modification [49]. We injected 5 mL of normal saline solution into the abdominal cavity of each sacrificed mouse and aseptically collected the peritoneal fluid as soon as possible and washed it three times to prepare the macrophages. We seeded 100 µL macrophages of 5 × 10^6^ cells which were counted by a standard medical blood count board into a 96-well cell culture plate (Sigma, Mendota Heights, MN, USA) and incubated them with RPMI 1640 medium for 3 h at 37 °C in a humidified 5% CO_2_ incubator. Following the attachment of the macrophages, we added 200 µL of 0.073% (m/v) neutral red into each well and incubated them for another 3 h, washed the plate three times, and subsequently added 100 µL/well of cell lysis solution (anhydrous ethanol:acetic acid = 1:1, *v*:*v*), which was then left overnight at 4 °C. We represent the pinocytosis of the peritoneal macrophage by the absorbance value at 550 nm of the neutral red ingested by the macrophages [50].

We used the freshly prepared splenic NK cells as effector cells and the H22 cells as target cells. We incubated the NK cells isolated by NK cell isolation kit II (Miltenyi Biotec, Bergisch Gladbach, Germany) according to the manufacturer’s instructions and H22 cells with RPMI 1640 medium at a ratio of 20:1 in 96-well plates. After incubation for 48 h, we determined the optical density (OD) by MTT assay using a microplate reader (Model 680, Bio-Rad, Hercules, CA, USA). We then calculated the percentage of NK cells using the following equation: 

NK cytotoxic activity (%) = (OD_0_ + OD_1_ − OD_2_)/OD_0_ × 100, where OD_0_ is the OD value of target cells, OD_1_ is the OD value of effector cells, and OD_2_ is the OD value of the effector-to-target cells. 

### 4.9. Assessment of Lymphocyte Subsets in Peripheral Blood

We analyzed the lymphocyte subsets in the peripheral blood using flow cytometry according to a previous method [51]. 24 h before the last dose, we collected blood (20 µL/mouse) from the caudal veins of living Kunming mice, and stained the cells with monoclonal antibodies (mAb) against CD8-FITC and CD4-PE for 30 min on ice in the dark. Then, we obtained the fluorescence-labeled lymphocytes by removing the erythrocytes. Finally, we resuspended the lymphocytes and measured the antigen expression using flow cytometry (BD FACSCallibur, BD, Franklin Lakes, NJ, USA). We analyzed the results using CellQuest Pro software and expressed them as the percentage of positive cells in the total amount of detected leukocytes.

### 4.10. Statistical Analysis

Here, we present all values as the mean ± standard deviation (S.D.) and we determined the statistical significance of differences using the student’s *t*-test and one-way analysis of variance. We consider the data to be significant at *p* < 0.05.

## 5. Conclusions

In conclusion, we used cold-water extraction and DEAE-cellulose 52 and G-100 column purification to obtain cAMPs-1A from *A. membranaceus* as a neutral polysaccharide (92.00%). Our HPGPC, IR, and IC results showed cAMPs-1A to be a pyranose containing α-type glycosidic linkages with a molecular weight of 1.23 × 10^4^ Da, which is mainly composed of glucose and small amounts of fucose, arabinose, galactose, and xylose. The in vivo antitumor and immunomodulatory effects of cAMPs-1A suggested that cAMPs-1A could significantly inhibit tumors growth and improve body immunity via the promotion of macrophage pinocytosis, the NK cells killing activity, and the percentages of T lymphocytes subsets in peripheral blood of tumor-bearing mice. This study outlines the strong in vivo antitumor and immunoregulation activities of cAMPs-1A with little side effect, which represent potentially useful medical functions in the prevention and treatment of HCC.

## Figures and Tables

**Table 1:** Inhibitory effect of cAMPs-1A on the immune organs indexes and growth of H22 tumor.

Treatment	Dose (mg/kg)	Thymus Index (mg/g)	Spleen Index (mg/g)	Tumor Weight (g)	Inhibitory Rate (%)
Blank group	-	3.30 ± 0.66	4.52 ± 0.47	-	-
Model group	-	1.51 ± 0.17 ^b^	7.93 ± 0.46 ^b^	2.63 ± 0.24	-
5-Fu	30	1.21 ± 0.11 ^a^	3.87 ± 0.15 ^a^	1.41 ± 0.13 ^a^	46.39
cAMPs-1A group	75	1.89 ± 0.16 ^a^	6.45 ± 0.32 ^a^	2.09 ± 0.16 ^a^	20.53
cAMPs-1A group	150	2.47 ± 0.21 ^a^	5.89 ± 0.40 ^a^	1.67 ± 0.14 ^a^	36.50
cAMPs-1A group	300	3.08 ± 0.36 ^a^	4.62 ± 0.29 ^a^	1.46 ± 0.11 ^a^	44.49

Note: ^a^ *p* < 0.05 compared to model group; ^b^ *p* < 0.05 compared to blank group.

**Table 2:** Effects of cAMPs-1A on the distribution of T cells subsets in peripheral blood.

Groups	Dose (mg/kg)	CD4^+^ (%)	CD8^+^ (%)
Blank group	-	37.66 ± 2.68	18.25 ± 1.40
Model group	-	29.22 ± 2.61 ^b^	15.26 ± 1.38 ^b^
5-Fu	30	28.43 ± 2.24	14.84 ± 1.41
cAMPs-1A groups	75	31.43 ± 3.11	16.26 ± 1.12
	150	33.79 ± 2.70 ^a^	17.83 ± 0.93 ^a^
	300	37.24 ± 3.74 ^a^	19.63 ± 3.42 ^a^

Note: ^a^ *p* < 0.05 compared to model group; ^b^ *p* < 0.05 compared to blank group.
